# Soil Property and Plant Diversity Determine Bacterial Turnover and Network Interactions in a Typical Arid Inland River Basin, Northwest China

**DOI:** 10.3389/fmicb.2019.02655

**Published:** 2019-11-26

**Authors:** Wenjuan Wang, Jianming Wang, Ziqi Ye, Tianhan Zhang, Laiye Qu, Jingwen Li

**Affiliations:** ^1^College of Forestry, Beijing Forestry University, Beijing, China; ^2^Research Center for Eco-Environmental Sciences, Chinese Academy of Sciences, Beijing, China

**Keywords:** arid land, inland river basin, bacterial β-diversity, bacterial network interactions, plant community, soil property

## Abstract

Water sources from the lower reaches of the Heihe River northwest China, located in an arid area impacted by environmental stresses, have promoted changes to the local soil and plant conditions; however, our understanding of variations and drivers of soil bacterial communities in an arid inland river basin remains unclear. Therefore, we collected 39 soil samples from a riparian oasis zone (ROZ) to the circumjacent desert zone (CDZ) at the lower reaches of Heihe River to evaluate bacterial communities based on the 16S rRNA gene data. We found that the bacterial community composition differed between ROZ and CDZ habitats, with significantly higher relative abundance of the phyla Gemmatimonadetes and Acidobacteria in ROZ, whereas the abundance of the phyla Actinobacteria and Deinococcus–Thermus was greater in CDZ. The difference in the bacterial community was almost entirely generated by the species turnover rather than the nestedness among all samples. In addition, we found that bacterial α-diversity index showed no significant difference between ROZ and CDZ habitats. The distance-decay analysis showed that spatial distance, plant community, soil property, and plant functional trait were correlated with bacterial community variations. However, the variation partition analysis (VPA) revealed that both soil properties and plant community strongly explained the difference [such as soil water content (WC), soil silt content, and plant community structure] compared with plant functional traits in bacterial β-diversity and species turnover. Based on a co-occurrence network analysis, we found that the bacterial network of ROZ, which had more negative correlations, higher average connectivity, shorter average path length, and smaller modularity, was more complex than the network of CDZ. This suggested that the bacterial community was more stable and less vulnerable to change in the ROZ habitat than in the CDZ habitat. Overall, our findings suggest that the heterogeneity of soil properties and plant community collectively affect the structure of the soil bacterial community in an arid inland river basin. However, the influence of plant functional traits on the variation of the bacterial community depends on soil properties and plant community.

## Introduction

Soil microbial communities play a critical role in regulating the functions and stability of an ecosystem (Bardgett and van der Putten, 2014; Glassman et al., 2018; Mori et al., 2018). Improving our knowledge of existing biogeographical patterns of soil microbes could provide opportunities to better understand ecosystem dynamics and functions under changing environmental condition. α-Diversity and β-diversity are two primary measures to investigate soil microbial distribution patterns. α-Diversity describes the number of species or taxa in a sampling site or habitat, whereas β-diversity shows the dissimilarity or alteration of species composition among different sites or habitats (Whittaker et al., 2001), correlated with species turnover and nestedness components (Baselga, 2010; Legendre, 2014). The turnover component is the replacement of some species by others from site to site, and nestedness mainly explains species loss, for example, some unique species existed in the richest site but were not present in the poorest site (Legendre, 2014). Consideration of both the species turnover and nestedness aspects of β-diversity separately can provide insight into environment variations and spatial distance to determine the groups of organisms within a system.

Distribution patterns and drivers of soil microbes have attracted extensive attention over the past several years. Previous studies have provided evidences that environmental heterogeneity could drive the composition and distribution of the microbial community (Maestre et al., 2015; Chai et al., 2019; Vieira et al., 2019; Zhang L. et al., 2019). For instance, Zhang K. et al. (2019) confirmed that differences in salinity negatively correlated with the similarity of the microbial community in a desert ecosystem, and the composition of the bacterial community converged on salt-tolerant species with increasing habitat salinity (Rath et al., 2019). In addition, some studies have indicated that soil water content (WC; Zhang et al., 2013; Maestre et al., 2015) and soil texture (Hu et al., 2014) have also affected a shift in the microbial community composition. Plant attributes, including plant diversity and functional traits, may be another factor that regulates microbes directly by determining the quality and quantity of leaf litter and root exudates or indirectly by influencing soil physiochemical properties (Soussi et al., 2016; Chai et al., 2019). However, it is still unclear how soil properties and plant attributes simultaneously drive the local assemblage and distribution of microorganisms.

In addition to the environmental factors, microbial interaction is another selection force that may contribute to microbial community assembly. Thus, clarifying their interactions can provide insight into microbial diversity and function (Hunt and Ward, 2015; Zhang Q. et al., 2018). Ecological networks, in which species are linked by positive (e.g., mutualism) and negative (e.g., competition) interactions, have been widely used in various ecosystem and microbial research (Zhou et al., 2011; Barberan et al., 2012; de Vries et al., 2018; Zheng et al., 2018). Although the network connections of species only represent co-occurrence of these species, not necessarily their direct physical interactions (Zhou et al., 2011), they also may reflect patterns that may not be detected by α/β-diversity (Barberan et al., 2012). For example, we can predict ecosystem stability and ecosystem response to disturbance based on topological properties and complexity of the networks (Deng et al., 2012). Recent studies have shown that drought causes greater changes to the co-occurrence networks of bacteria and greater instability in the soil bacterial community than in the fungal community in grassland mesocosms (de Vries et al., 2018). In addition, studies in semi-arid grassland soils also confirmed that the microbial network becomes more complex and interlinked with the increase in precipitation (Wang S. et al., 2018). Combined, these previous studies have significantly improved our understanding of patterns and drivers of microbes in arid land at the large scale. No study has been conducted to reveal the influences of an inland river on the composition and distribution of soil microbes at the local scale, although the oasis zone, shaped by an inland river, has higher biological diversity and plays an important role in arid land.

The Ejina Oasis in northwestern China, which plays an important ecological and economic role in extremely arid land yet is vulnerable to anthropogenic disturbance, is shaped by the lower reaches of the Heihe River (Ding et al., 2017). Thus, two significant areas, a riparian oasis zone (ROZ) and circumjacent desert zone (CDZ), have formed in the small geographic area of the Ejina Oasis. However, these two habitats were significantly influenced by changes in the quantity of water sources and exhibited mutual transformation with desertification or rejuvenation of the oasis (Zhang M. et al., 2018), which may further result in substantial changes of the soil bacterial community. Indeed, plant communities are significantly different between the ROZ and CDZ (Jin et al., 2010). For instance, plant communities in the ROZ are dominated by the tree species *Populus euphratica*, with sparse understory vegetation (e.g., *Tamarix ramosissima*, *Sophora alopecuroides*), whereas communities in the CDZ are mainly dominated by xeric shrubs, such as *Reaumuria soongarica* and *Nitraria tangutorum*. Thus, this condition provided a unique opportunity to investigate the soil bacterial community under different dominant vegetation in the same climatic conditions. In addition, soil environmental conditions may lead to a change in the soil bacterial community (Wang et al., 2019; Zhang K. et al., 2019), as some studies have clearly shown the significant spatial heterogeneity of soil properties in these habitats (e.g., soil WC, salinity, and particle composition), further influencing the distribution of plant species (Ding et al., 2017; Zhao et al., 2017). Bacteria play an important role in ecosystem health and are directly involved in energy conversion, element transformation, and nutrient biogeochemical cycling processes; however, few studies have focused on elucidating the turnover and drivers of the bacterial community across an arid inland river basin of northwest China.

To explore the spatial turnover and interaction of the bacterial community in the arid inland river basin, 39 soil samples were collected from the ROZ to the CDZ at the lower reaches of Heihe River, Inner Mongolia, China, and used to determined bacterial communities based on 16S rRNA. Using these data, we aimed to answer the following questions: (1) How do the diversity and composition of the bacterial community change vertical to the arid inland river and what is the relative contribution of the turnover and nestedness to the bacterial β-diversity? (2) How do the plant attributes, soil properties, and spatial factors drive the biogeographical distribution of bacteria? and (3) What is the difference in the co-occurrence networks of the bacterial community between the ROZ and the CDZ?

## Materials and Methods

### Study Site and Field Sampling

**FIGURE 1:**
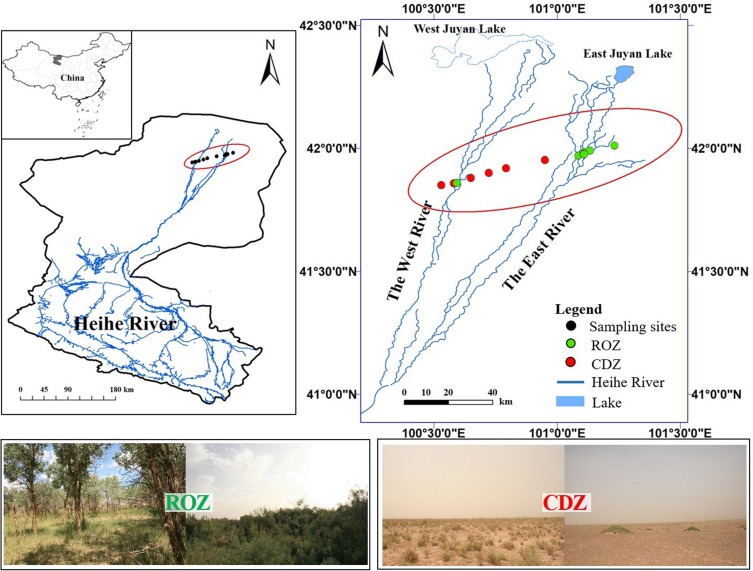
Location of sampling sites and study area. The data of Heihe River was provided by Cold and Arid Region Science Data Center at Lanzhou (http://westdc.westgis.ac.cn). The pictures showed typical vegetations from riparian oasis zone (ROZ) and circumjacent desert zone (CDZ) environments.

The study was conducted during the peak growing season (July) in the lower reaches of the Heihe River in Ejina Banner, Inner Mongolia (101°05′E–101°13′E, 41°57′N–42°00′N), which is a typical arid inland river basin in northwest China (including a typical ROZ and CDZ). The sampling area is characterized by an arid continental climate, with hot summer and cold winter. The mean annual temperature is 8.4°C, and the mean temperature is 26.4°C in July, with the extremely highest temperature reaching 43.1°C and daily temperature range reaching 14–20°C, whereas the mean temperature is −11.9°C in January, with the extremely lowest temperature reaching −37.6°C. The mean annual precipitation is approximately 37.9 mm, which mostly occurs during June to August, but the precipitation is almost unavailable because each precipitation is less than 5 mm. In addition, the annual potential evaporation is approximately 100 times the annual precipitation. The 13 sample sites, seven sites from ROZ and six sites from CDZ, were selected from the ROZ to the CDZ to cover the main typical vegetation types of this area (Figure 1).

At each site, three representative plots (20 m × 20 m) were established as replicates. Vegetation communities were investigated and recorded. In addition, the geographical coordinates of each plot were recorded using a GPS (GPSmap 629sc). Leaves of dominant plant were collected and stored at 4°C to later determine the plant functional traits, such as leaf thickness (LT). The soil sample of each plot was collected by randomly selecting 20 cores (0–15 cm depth) in each plot that were thereafter well-mixed and homogenized. In total, 39 soil samples were collected in this study. After removing and discarding most leaves and stones with a 2-mm mesh screen, the soil samples were divided into two portions: one portion was used for the physicochemical analysis, and the other portion was stored at −20°C for DNA extraction.

### Analysis of Soil and Plant Characteristics

Soil organic carbon (SOC), total nitrogen (TN), total phosphorus (TP), and available nitrogen (AN) were measured following the procedures described by Wang et al. (2017a). Soil WC was gravimetrically determined after drying the soil in an oven at 105°C for 48 h. Soil electrical conductivity (EC) and pH were measured by a conductivity meter (DDS-307A, REX, Shanghai) and digital pH meter (PHS-3E, REX, Shanghai; 1:5 w/v), respectively (Rath et al., 2019). In addition, soil particle composition was analyzed using a Mastersizer 2000 laser particle size analyzer (Malvern Instruments, United Kingdom) that divided the soil into sand (>50 μm), silt (2–50 μm), and clay (<2 μm).

Leaves were collected for measuring plant functional traits from 5 to 10 randomly selected plants of each dominant species in each plot. Specific leaf area (SLA) was measured as the ratio of leaf surface area to dry mass. Leaf dry matter content (LDMC) was calculated as the ratio of leaf dry mass to fresh mass. LT was measured with Digital Vernier Caliper (PD-151, Prokit’s Industries Co., Ltd., China). Leaf carbon content (LCC) was determined using K_2_Cr_2_O_7_ oxidation method (Magdoff et al., 1996). Leaf nitrogen content (LNC) and leaf phosphorus content (LPC) were respectively determined by Kjeldahl procedure (Michałowski et al., 2013) and ammonium molybdate spectrophotometric method (Bowman, 1988) following H_2_SO_4_–H_2_O_2_ digestion. Then, we calculated the community levels’ functional trait index by the sum of dominant species content contribution divided by relative abundance. Mean values for soil properties and community functional trait indexes at ROZ and CDZ habitats are presented in Supplementary Table S1.

### 16S rRNA Gene Amplicon Sequencing and Bioinformatics Analysis

Soil DNA was extracted from 0.25-g soil samples within 1 week after storage for a week using PowerSoil DNA Isolation Kit (MoBio Laboratories, Carlsbad, CA, United States) according to the manufacturer’s instructions. The V3-V4 hypervariable regions of bacterial 16S rRNA genes were amplified using the forward primer 338F (5′-ACTCCTACGGGAGGCAGCAG-3′) and the reverse primer 806R (5′-GGACTACHVGGGTWTCTAAT-3′) (Mwaheb et al., 2017; Li et al., 2018), which include an Illumina adapter sequence and a bar code sequence unique to each sample (provided by Allwegene Technology, Beijing). PCR was conducted in triplicate with an ABI GeneAmp 9700 PCR in 25-μl reaction volumes, containing 12.5 μl 2 × Taq PCR MasterMix, 3 μl bovine serum albumin (BSA) (2 ng/μl), 2 μl primer (5 μM) (Biomed, China), 2 μl (30 ng) template DNA, and 5.5 μl ddH_2_O. The cycling conditions were 95°C for 5 min; followed by 32 cycles of 95°C for 45 s, 55°C for 50 s, and 72°C for 45 s; and a final extension at 72°C for 10 min. Three replicate amplifications were mixed together, purified using an Agencourt AMPure XP Kit (Beckman Coulter, United States) following the manufacturer’s instructions and quantified using Caliper (Caliper Gene 760517, United States). Finally, purified amplicons were pooled in equimolar solution and paired-end sequenced (2 × 300) using a Miseq Reagent Kit v3 (Illumina, United States) on an Illumina Miseq platform by Allwegene Technology (Beijing).

The sequence data were processed using the QIIME packages (quantitative insights into microbial ecology, v1.2.1; Caporaso et al., 2010). After identifying and removing the chimeric sequences with USEARCH, the remaining sequences were clustered into operational taxonomic units (OTUs) with 97% identity threshold using UCLUST, and singleton OTUs (with only one read) were removed. The taxonomy of each OTU was assigned through the RDP Classifier against the Silva128 16S rRNA database, and only OTUs annotated as bacteria were maintained for further analysis. The OTU table was rarefied to 18,182 sequences per sample to correct differences in sequencing depth.

The bacterial DNA sequences in our study have been submitted to SRA in the NCBI database under accession number SRP200254.

### Network Construction and Analysis

Networks were constructed based on 16S rRNA sequence data for ROZ and CDZ habitats separately. The process can be divided into four steps: sequence data collection, data transformation, pairwise similarity matrix calculation, and adjacent matrix determination (Zhou et al., 2010, 2011; Deng et al., 2012). Specifically, for each habitat, only OTUs occurring in half of the total samples were used to construct the network. The abundance of OTUs was log_10_-transformed, and missing values were filled with 0.01 if paired valid values were available. Then, the rest of the analysis was carried out using the default setting of the Molecular Ecological Network Analysis pipeline^1^; and the resulting graphs were depicted using Cytoscape v.3.6.1 software (Shannon et al., 2003).

Differences of global network properties and modularity between ROZ and CDZ habitats reflect different bacterial relationships and ecosystem stability. The differences of the network indexes were compared using a statistical Z-test between molecular ecological network (MEN) and random networks. A Student *t*-test was conducted to compare these differences between ROZ and CDZ networks.

### Statistical Analysis

The dominant phyla and genera of the bacterial community were identified based on a relative abundance of greater than 1% among all samples or in ROZ and CDZ, respectively. Analysis of variance (ANOVA) was performed to evaluate significant differences in dominant phylotypes and α-diversity between ROZ and CDZ using IBM SPSS Statistics for Windows (Version 23.0, IBM Corp., Armonk, NY, United States). The data that were non-normal or heterogeneity of variance were log or square root transformed before analyses. Otherwise, we performed non-parametric Kruskal–Wallis tests. Pearson correlation analysis was used to test the relationships between α-diversity and soil and plant properties.

The overall variation in bacterial community was depicted by non-metric multidimensional scaling analysis (NMDS) (Kruskal, 1964), while the similarity between ROZ and CDZ was tested with ANOSIM and PERMANOVA methods in the “vegan” package of R (Oksanen et al., 2017). Pairwise spatial distance was measured with latitudinal and longitudinal coordinates using the “fossil” package (Vavrek, 2015), and soil property distance and plant functional trait distance were calculated based on standardized soil properties and plant functional traits. Matrices of pairwise plant community dissimilarity were analyzed using the Bray–Curtis method with plant relative abundance data. For less extreme distributions, logarithmic transformation was conducted prior to calculating the pairwise community Bray–Curtis distance of bacteria within the “vegan” package (Oksanen et al., 2017); β-diversity (d_BC_) was separated into species turnover (d_BC–bal_) and nestedness (d_BC–gra_) using the “betapart” package of R (Baselga and Orme, 2012; Baselga et al., 2018). Distance-decay curves were depicted between bacterial community dissimilarity and spatial distance, plant community dissimilarity, plant functional trait distance, or soil property distance.

Mantel and partial-Mantel tests were used to examine the influence of spatial distance, plant functional trait distance, plant community dissimilarity, and soil property distance on the β-diversity of the bacterial community and its components (10,000 permutations) (Oksanen et al., 2017). Then, we conducted a principal coordinate analysis (PCoA) based on the Bray–Curtis distance to acquire the plant community and bacterial community vectors (Oksanen et al., 2017). To reduce the dimensionality, only the first six vectors of plant community composition, which represented more than 70% of the total variation, were used in the following analysis (Wang J. et al., 2018). These six vectors combined with plant richness (PR) were prepared as plant community data to participate in a variation partition analysis (VPA). Next, we used principal coordinates of neighbor matrices (PCNM) to obtain the spatial variables (Borcard and Legendre, 2002), and only positive PCNM vectors were used for the VPA. Before conducting the VPA, spatial variables, plant community factors, plant functional trait factors, and soil properties were subjected to forward selection until *P* was <0.05 and the VIF value was <10 using the “packfor” package (Dray et al., 2009). Finally, the relative contribution of spatial distance (three positive PCNM vectors), plant community factors (five PCoA variables and PR), plant functional trait factors (SLA, LDMC, LCC, LNC, LPC), and soil properties (AN, pH, EC, WC, clay, and silt) on bacterial community β-diversity and its components was calculated by VPA (Mcardle and Anderson, 2001). In addition, we also calculated the single contribution of variables in plant community factors and soil properties separately after controlling for the other variables.

## Results

### Bacterial Community Composition and α-Diversity

After removing the archaea and singleton OTUs, the number of bacterial sequences per sample ranged from 18,182 to 82,732, and these were classified as 707 to 2,477 OTUs. When all samples were compared, the OTU table was rarefied to 18,182 sequences per sample with an OTU richness from 582 to 1,955.

**FIGURE 2:**
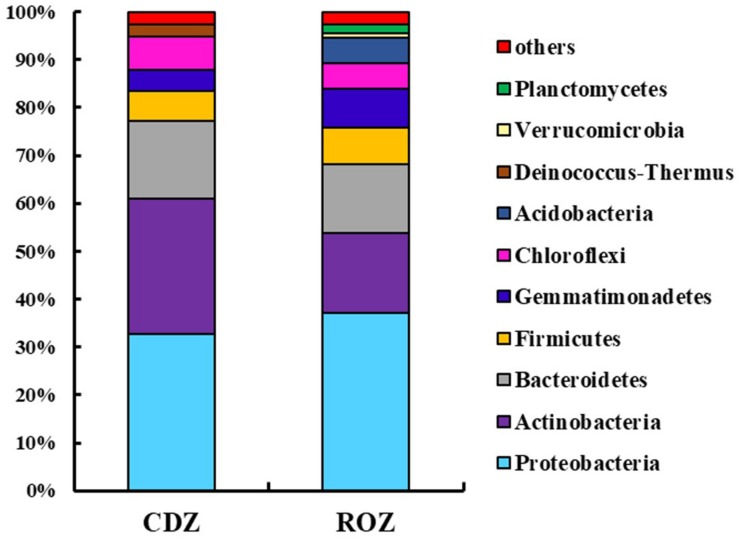
Mean relative abundances of dominant bacterial phyla in different habitats. CDZ, circumjacent desert zone; ROZ, riparian oasis zone.

Across all samples, the predominant phyla, Proteobacteria, Actinobacteria, Bacteroidetes, Firmicutes, Gemmatimonadetes, Chloroflexi, Acidobacteria, Deinococcus–Thermus, and Planctomycetes (relative abundance >1%, Supplementary Figure S1), accounted for more than 95% of the bacterial sequences. And the percentage of some predominant phyla was specifically different between the ROZ and CDZ. Specifically, the relative abundances of Actinobacteria and Deinococcus–Thermus phyla were significantly higher in CDZ than in ROZ habitat, whereas Gemmatimonadetes, Acidobacteria, Verrucomicrobia, and Planctomycetes phyla were more abundant in ROZ than in CDZ habitat (Figure 2 and Supplementary Table S2). In addition, the relative abundances of dominant bacterial genera were also determined (relative abundance >1%, Supplementary Table S2). For example, dominant genera (such as *Nafulsella*, *Truepera*, and *Rubrobacter*), belonging to Bacteroidetes, Deinococcus–Thermus, and Actinobacteria phyla, were significantly higher in CDZ than those in ROZ habitat. In contrast, both *Marinobacter* and *Marinimicrobium* of the phylum Proteobacteria were more abundant in ROZ than in CDZ habitat (Supplementary Table S2).

One-way analysis of variation showed that bacterial Shannon index was not significantly different between ROZ and CDZ habitats (*P* > 0.05, Supplementary Table S3). The index was significantly negatively correlated with EC and soil TP and positively correlated to soil silt content (Supplementary Table S4). In contrast, there was no significant correlation between the index and soil WC and PR (*P* > 0.05, Supplementary Figure S2).

### Bacterial Community Structure and β-Diversity

**FIGURE 3:**
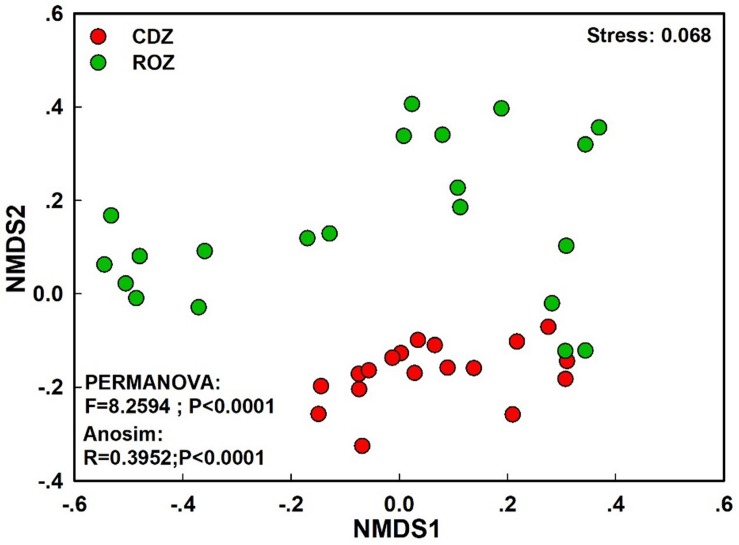
Non-metric multidimensional scaling ordination (NMDS) of the bacterial community. CDZ, circumjacent desert zone; ROZ, riparian oasis zone.

**FIGURE 4:**
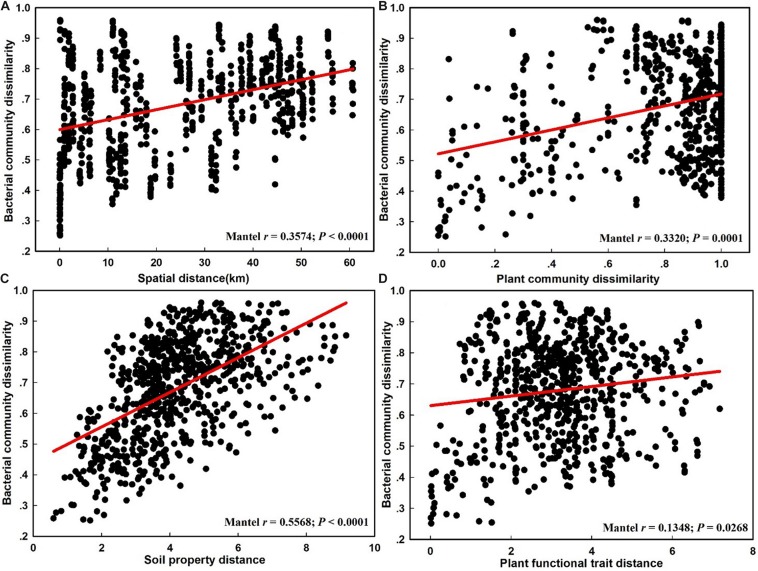
The relationship between the bacterial community dissimilarity and spatial distance **(A)**, plant community dissimilarity **(B)**, soil property distance **(C)**, and plant functional trait distance **(D)**.

The NMDS plot based on the Bray–Curtis distance showed that the structure of the bacterial community was significantly different between the CDZ and ROZ habitats (ANOSIM, *R* = 0.3952, *P* < 0.0001; PERMANOVA, *F* = 8.2594, *P* < 0.0001; Figure 3). Furthermore, the Bray–Curtis distance of the bacterial community across 39 soil samples was significantly correlated with spatial distance (*r* = 0.3574, *P* < 0.0001; Figure 4A), which indicated a spatial pattern of bacterial community structure along the ROZ to the CDZ.

When we separated the β-diversity into two components, the species turnover nearly accounted for the entire bacterial β-diversity (98.3%) rather than nestedness (1.7%, Supplementary Table S5). Moreover, we also found that species turnover of the bacterial community was positively correlated with the spatial distance (*r* = 0.3626, *P* < 0.0001), whereas bacterial nestedness showed no significant relationship with spatial distance (*P* > 0.05, Table 1).

**TABLE 1:** The relationship among bacterial β-diversity, turnover and nestedness and soil property distance, plant community dissimilarity, spatial distance, and plant functional trait using Mantel and partial Mantel tests.

**Effects of**	**Controlling for**	**d_BC_**	**d_BC–bal_**	**d_BC–gra_**
Soil property distance		0.5568^∗∗∗^	0.5715^∗∗∗^	−0.2109
Soil property distance	Plant community dissimilarity	0.5293^∗∗∗^	0.5460^∗∗∗^	−0.2162
Soil property distance	Geographic distance	0.5055^∗∗∗^	0.5220^∗∗∗^	−0.1884
Plant community dissimilarity		0.3320^∗∗∗^	0.3051^∗∗∗^	−0.0038
Plant community dissimilarity	Soil property distance	0.2678^∗∗∗^	0.2339^∗∗∗^	0.0492
Plant community dissimilarity	Geographic distance	0.2213^∗∗^	0.1897^∗∗^	0.0492
Spatial distance		0.3574^∗∗∗^	0.3626^∗∗∗^	−0.0642
Spatial distance	Soil property distance	0.2692^∗∗^	0.2683^∗∗∗^	−0.0518
Spatial distance	Plant community dissimilarity	0.2850^∗∗^	0.2949^∗∗∗^	−0.1197
Plant functional trait		0.1348^∗^	0.1448^∗^	−0.0746

*d_*BC*_, β-diversity; d_*BC–bal*_, replacement component; d_*BC–gra*_, nestedness component. ^∗∗∗^P < 0.0001; ^∗∗^P < 0.001; ^∗^P < 0.05.*

The results from the Mantel test indicated that plant community dissimilarity and soil property distance were strongly related to the bacterial β-diversity and species turnover (*P* < 0.0001; Figure 4 and Table 1), whereas plant functional traits had a weaker influence on β-diversity and bacterial species turnover (*P* < 0.05; Figure 4 and Table 1). The partial-Mantel test revealed that plant community dissimilarity and soil property distance were still significantly correlated with bacterial β-diversity and species turnover when the other factors were controlled (*P* < 0.001, Table 1). However, no correlations were found between bacterial species nestedness and plant community dissimilarity, soil property distance, and plant functional traits (*P* > 0.05, Table 1).

### Drivers of Bacterial β-Diversity and Species Turnover

**FIGURE 5:**
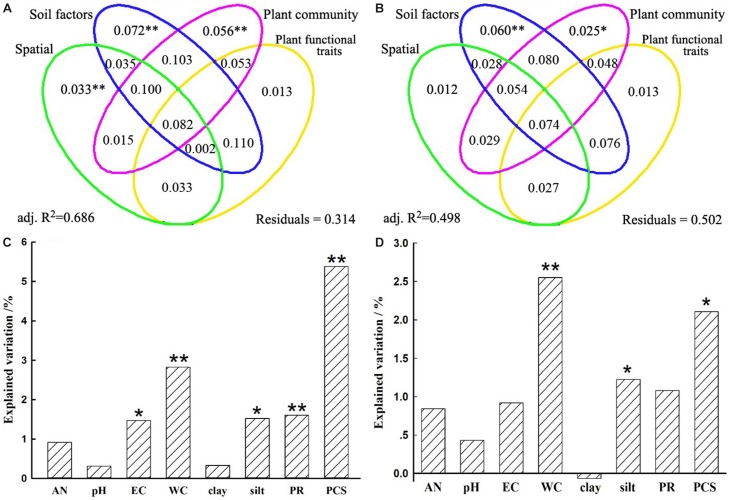
Variation partition analysis of the effects of spatial distance, soil properties, plant community factors, and plant functional traits on bacterial community β-diversity **(A)** and its turnover component **(B)**. The unique contribution of soil and plant community factors individually on bacterial community β-diversity **(C)** and its turnover component **(D)** after controlling for the other variables. Significance was determined by an ANOVA-like permutation test in CCA (Canonical Correspondence Analysis) with ^∗∗^ representing at 0.01 significance level while ^∗^ at 0.05 level. AN, available nitrogen; EC, electrical conductivity; WC, soil water content; clay, soil clay content; silt, soil silt content; PR, plant richness; PCS, plant community structure.

A VPA was performed to quantify the relative contributions of spatial distance, soil properties, plant community dissimilarity, and plant functional traits on bacterial β-diversity and species turnover. Spatial distance, soil properties, plant community dissimilarity, and plant functional traits separately explained 28.5, 49.6, 38.8, and 27.2% variations of the bacterial β-diversity and 20.5, 36.0, 28.4, and 21.0% variations of the bacterial species turnover, respectively (Supplementary Table S6). However, when other variables were controlled, the unique contribution of predictor variables differed remarkably. Generally, the unique contributions of soil properties on the bacterial β-diversity and species turnover (7.2 and 6.0%) were slightly more important than or as important as the contributions of plant community (5.6 and 2.5%) (Figure 5 and Supplementary Table S6). Although less important than soil and plant community factors, spatial variables also accounted for statistically significant fractions of variation in bacterial β-diversity (3.3%, *P* = 0.002), but this was not true for the species turnover component (1.2%, *P* = 0.143). Nevertheless, plant functional traits could not significantly explain variations in bacterial β-diversity (*P* = 0.114) or species turnover (*P* = 0.154). For these two models, the fractions representing shared effects were larger and explained 8.2 and 7.4% of the variations of bacterial β-diversity and species turnover, respectively (Figures 5A,B), indicating that all predictors simultaneously acted on the bacterial β-diversity or turnover component. In particular, the shared effects were mostly found among soil properties and plant community (Figure 5). For the β-diversity, the total variation explained by the models including all predictors was 68.6%. In contrast, the total variation explained by the models was 49.8% for the species turnover (Figure 5 and Supplementary Table S6).

Furthermore, soil WC and EC were more closely related to bacterial β-diversity (*r* = 0.4020, 0.4174; *P* = 0.0001; Supplementary Table S7), which variations could explain 17.3 and 16.0% variations of bacterial β-diversity (Supplementary Figure S2). Similar results can also be found on species turnover (Supplementary Table S7 and Supplementary Figure S2). In addition, under the condition of independent explanation of soil properties (7.2%), we found that EC, soil WC, silt could significantly explain 1.5, 2.8, and 1.5% variations of bacterial β-diversity, respectively (Figure 5C). In contrast, soil WC and silt could significantly explain 2.6 and 1.2% variations of species turnover under condition of 6.0% independent soil properties explanation (Figure 5D). These results suggested that soil properties collectively affected the bacterial communities, while soil WC and EC were relatively more important among these properties.

### Network Interactions of the Bacterial Community in ROZ and CDZ Habitats

**FIGURE 6:**
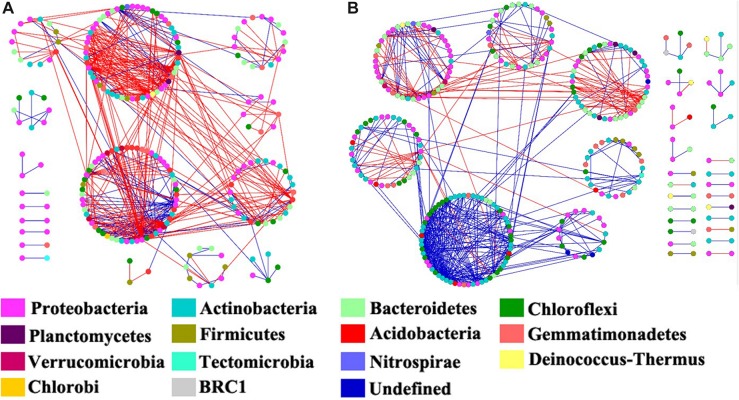
The co-occurrence network maps of bacterial community at operational taxonomic unit (OTU) level in riparian oasis zone ROZ **(A)** and circumjacent desert zone (CDZ) **(B)** soils. Colors of nodes represent different phyla. Red links indicated negative interaction between the two individual nodes, whereas blue links indicate positive interaction.

We constructed the bacterial networks at the OTU level to determine the co-occurrence patterns in the ROZ and CDZ habitats (Figure 6). The network topographical structure showed that the soil bacterial community had a higher average connectivity (avgK), shorter average path length (GD), and smaller modularity in ROZ soils than in CDZ soils (Table 2), suggesting that the bacterial network in ROZ soils was more complex than the CDZ network. A total of 479 links were identified in the ROZ network, and 584 links were identified in the CDZ network. In addition, the number of negative links in ROZ was significantly higher than that in the CDZ habitat (65.6% vs. 28.4%), suggesting that competition dominated in the ROZ habitat, while mutualistic relationships mainly existed in the CDZ habitat (Figure 6). More than 50% of the nodes in the two networks belonged to Proteobacteria and Actinobacteria phyla (Figure 6). Although the bacterial community structures and species interactions were greatly different between ROZ and CDZ habitats, we found 51 OTUs that were shared in the two networks, indicating that there were generalists that could adapt to various habitats (Supplementary Figure S3).

**TABLE 2:** Topological properties of the empirical ecological networks of bacterial communities in CDZ and ROZ and their associate random ecological networks.

**Community**	**Empirical networks**							**Random network**		
		
	**Similarity threshold (St)**	**Network size (*n*)**	**Links**	**Avg. connectivity (avgK)**	**Avg. path length (GD)**	**Ave. clustering coefficient (avgCC)**	**Modularity (no. of modules)**	**Avg. path length (GD)**	**Ave. clustering coefficient (avgCC)**	**Modularity (no. of modules)**
CDZ	0.86	321	584	3.639	5.041 a	0.091 a	0.663 a	3.878 ± 0.052	0.030 ± 0.006	0.517 ± 0.006
ROZ	0.88	220	479	4.355	4.057 b	0.040 b	0.530 b	3.427 ± 0.050	0.061 ± 0.010	0.440 ± 0.007

*The different letters at the same column indicated significant difference of the properties between CDZ and ROZ based on the Student’s *t*-test with standard deviations derived from corresponding random networks. CDZ, circumjacent desert zone; ROZ, riparian oasis zone.*

## Discussion

### Changes in the Composition of the Bacterial Community in an Arid Inland River Basin

Elucidation of the spatial variation in microbial taxonomic structure contributes to our understanding of microbial adaptation and response to alterations in biotic and abiotic environments (Chen et al., 2016). In the present study, we found differences in the composition of the bacterial community between the ROZ and CDZ in the lower reaches of the Heihe River basin in northwest China (Figure 2 and Supplementary Table S2). For example, the relative abundance of the phyla Actinobacteria and Deinococcus–Thermus phyla was significantly higher in CDZ than that in ROZ habitat, whereas Acidobacteria and Verrucomicrobia phyla were higher in ROZ than those in CDZ habitat. This finding was consistent with previous studies showing that Actinobacteria phylum was more abundant in more arid areas (Wang et al., 2015) and that soil aridity promoted the decrease of Acidobacteria in global drylands (Maestre et al., 2015), suggesting that water availability is an important factor affecting the composition of a bacterial community. Another possible reason for this difference is the varied adaptations and stress tolerance levels of species. For instance, Actinobacteria has higher ability to grow under salt concentrations and radiation (Mohammadipanah and Wink, 2016), while Verrucomicrobia dominated in prairie soils and was most abundant in intermediate temperature and precipitation conditions (Fierer et al., 2013). In addition, the relative abundance of Proteobacteria, which is a common member of communities comprising copiotrophic taxa, with quick response to high moisture and resource availability (Fierer et al., 2007), showed no significant differences between the ROZ and CDZ habitats. However, different Proteobacteria genera exhibited significant differences between the habitats (Supplementary Table S2). This suggests that the same phylum contains different genera that responded to different environmental factors.

Bacterial β-diversity across all samples was primarily generated by species turnover component rather than by species nestedness with 98% relative contribution (Supplementary Table S5), which is consistent with a recent study in a typical dryland (Wang et al., 2017b). In addition, we found that bacterial Shannon index showed no statistically significant difference between CDZ and ROZ habitats (Supplementary Table S3). These results indicate that changes in bacterial community composition may primarily arise from the replacement of some species rather than species loss or gain from ROZ to CDZ habitats.

### Drivers of Bacterial Community β-Diversity and Its Components

Along with remarkable differences in the composition of the bacterial community between ROZ and CDZ habitats, we found that the dissimilarity of the bacterial community increased with the increasing spatial distance, implying changes in the bacterial communities (Figure 4). The changes could be caused by biotic and/or abiotic conditions at the local scale (Pajares et al., 2016; Dukunde et al., 2019; Vieira et al., 2019). Indeed, we found a significant relationship among soil properties, plant community, plant functional traits, and bacterial community dissimilarity (Figure 4).

Among biotic factors, we found that PR and community structure, especially the latter factor, significantly influenced the β-diversity of the bacterial community, as PR and community structure purely explained 1.6 and 5.4% of variation in bacterial community structure, respectively (Figure 5). Similar results were also found in temperate deciduous forests (Dukunde et al., 2019) as well as subtropical forests (Chen et al., 2019), which indicated plant-specific characteristics may have a more important effect than the number of species on bacterial community variations. Generally, specific plants offer unique carbon resources *via* plant leaf litter, fine root, or root exudates, and this supply makes the bacterial community distinctly plant dependent (Hooper et al., 2000; Millard and Singh, 2010; Eisenhauer et al., 2011). However, plant functional traits exerted no significantly unique influence on bacterial β-diversity in our study (Figure 5). In contrast, the findings of a previous study reported that plant functional traits were significantly related to the soil bacterial community composition during secondary forest succession on the Loess Plateau, China (Chai et al., 2019). This difference may be because plant leaf functional traits affected bacterial community depending on the other factors, such as soil properties. Indeed, we found that plant leaf functional traits had more shared explanation with soil properties on bacterial β-diversity and bacterial turnover (11 or 7.6%; Figures 5A,B).

In this study, soil properties also significantly explained variations of bacterial communities. Here, we found that soil WC, EC, and soil silt content was relatively more important among all soil properties. This is not surprising because water availability is one of the most important factors that constrain the activity of soil microorganisms, especially in arid land (Wang S. et al., 2018). Several previous studies have also shown a strong influence of water availability on bacterial community variations, with bacterial dissimilarity being generally higher with increasing precipitation distance on dryland (Nielsen and Ball, 2015; Wang et al., 2015). Furthermore, in arid land, the pressure of water availability is often accompanied by increased salinity content owing to rapid evaporation, leading to the accumulation of salinity (Bachran et al., 2018; Zhao et al., 2019). In this regard, some studies have reported that salinity exerts a strong selection pressure on the microbial community, resulting in deterministic processes in microbial assembly (Rath et al., 2019; Zhang K. et al., 2019). A similar phenomenon was also found in our study, indicating that bacterial richness significantly decreased with increasing EC, while bacterial community dissimilarity increased along EC distance (Supplementary Figure S2). This observed trend might be attributed to the fact that many microorganisms fail to adapt to osmotic stress induced by elevated salinity content, resulting in the death or inactivity (Campbell and Kirchman, 2013; Oren, 2015), thus reducing the bacterial richness and altering the composition and structure of bacterial communities. Compared to soil WC affecting the bacterial community *via* directly changing the water availability or indirectly adjusting the carbon availability, soil structure could also exert influences on the bacterial community. Better soil structure could enhance the water holding capacity and substrate availability, integrating the underground environment and thus providing an appropriate habitat for microorganisms (Hu et al., 2014). Notably, soil silt content significantly influenced the bacterial β-diversity by explaining the unique 1.5% of the variation observed in our study (Figure 5). Similar results were also found in Tibetan alpine grasslands, specifically, soil microbiomass was positively correlated with the silt content but negatively correlated with the sand content (Chen et al., 2016).

In addition, bacterial β-diversity and species turnover could be attributed to a combination of factors associated with soil properties and plant in our study (Figure 5). This result was consistent with a study in Yellow River Delta that indicated that the shift of bacterial community along a salinity gradient was mainly because of the combination of environment and plants (Li et al., 2018). These results suggested that water supplemented from the lower reaches of the Heihe River in arid land would sharply change the soil environment and plant community (Xi et al., 2016; Ding et al., 2017; Zhao et al., 2019), thus forming a ROZ that will further influence the bacterial community.

We also found a significantly unique effect of spatial distance on bacterial β-diversity, which is generally explained by dispersal limitation (Wang et al., 2015; Mori et al., 2018); however, given the scale of our study, such influence may have resulted from the heterogeneity of other unconsidered factors.

### Effects of an Arid Inland River on Bacterial Network Interactions

Water from the inland river played a key role in the arid land by altering the relative abundances and composition of organisms and by changing the interactions among species (such as competition and mutualism) (Hunt and Ward, 2015). Revealing the co-occurrence of bacteria will provide insight into the variations of the bacterial community in different habitats (Wang S. et al., 2018). Higher average connectivity and shorter average path length have been found in ROZ than CDZ, suggesting a more complex co-occurrence network in ROZ habitat, which is similar to a recent study showing that the microbial network became more complex with the increase in precipitation in water-limited ecosystems (Wang S. et al., 2018). This indicates that bacterial communities in a water-limited desert are more unstable and vulnerable to change. In addition, more negative connections were observed in ROZ than CDZ habitat (65.6% vs. 28.4%, respectively), partly owing to the increased water and nutrient availability stimulated by the supply of water from the inland river, providing more opportunities for different species to interact with each other (Tian et al., 2018; Wang S. et al., 2018). Together, the results mentioned above revealed that river-induced environmental heterogeneity may influence interactions among bacterial networks.

## Conclusion

This study confirmed that bacterial community composition was significantly altered from the ROZ to the CDZ, which was attributed to the species turnover rather than species nestedness in an arid region. This result was significantly determined by the heterogeneity of soil properties and plant community factors due to supplementary water from an inland river; plant leaf functional traits explained less of the variations of the bacterial community composition. In addition, the bacteria network became more stable and complex from the CDZ to the ROZ. Our study enhances the understanding of the relationships between bacterial community composition and water resources-induced environmental changes owing to the lower reaches of the Heihe River. In addition, this study provides a scientific basis for predicting responses of microbial communities to future climate change in arid land. Future studies are necessary to investigate the influence of inland river on temporal dynamics of microbial communities and their networks due to seasonal dynamics of river water.

## Data Availability Statement

The datasets generated for this study can be found in the SRA of NCBI database under accession number SRP200254.

## Author Contributions

WW, JL, and JW designed the study. WW, ZY, and TZ performed the field investigation and collected the data. WW and JW conducted the statistical analysis. WW and LQ wrote the manuscript.

## Conflict of Interest

The authors declare that the research was conducted in the absence of any commercial or financial relationships that could be construed as a potential conflict of interest.

## Footnotes

1 http://ieg4.rccc.ou.edu/mena
