# Supplementary material for: Soil Property and Plant Diversity Determine Bacterial Turnover and Network Interactions in a Typical Arid Inland River Basin, Northwest China
Source: Front Microbiol. 2019 Nov 26;10:2655. doi: 10.3389/fmicb.2019.02655 (PMC6888015; doi:10.3389/fmicb.2019.02655)
Supplement: Supplementary file 1 [file Data_Sheet_1.docx]

**Soil Property and Plant Diversity Determine Bacterial Turnover and Network Interactions in a Typical** **Arid Inland River Basin, Northwest China**

**Wenjuan Wang^1^, Jianming Wang^1^, Ziqi Ye^1^, Tianhan Zhang^1^, Laiye Qu^2^, Jingwen Li^1*^**

^1^College of Forestry, Beijing Forestry University, Beijing 100083, China

^2^Research Center for Eco-Environmental Sciences, Chinese Academy of Sciences, Beijing 100085, China

***Correspondence:**

Jingwen Li

lijingwenhy@bjfu.edu.cn

| **Table S1. Soil properties and plant characteristics of ROZ and CDZ habitats** | | | | | | | | |
| --- | --- | --- | --- | --- | --- | --- | --- | --- |
|  | **SOC**  **(g/kg)** | **TN**  **(g/kg)** | **AN**  **(mg/kg)** | **TP**  **(g/kg)** | **pH** | **LT**  **(mm)** | **LCC**  **(g/kg)** | **LNC**  **(g/kg)** |
| ROZ | 7.17±3.27 a | 0.70±0.29 a | 38.95±20.99 a | 0.57±0.10 b | 8.84±0.20 a | 0.35±0.07 | 427.55±52.42 | 26.10±6.65 |
| CDZ | 3.33±1.82 b | 0.38±0.13 b | 10.69±6.10 b | 0.65±0.08 a | 8.45±0.21 b | 0.43±0.18 | 400.80±52.92 | 25.46±6.49 |
|  | **EC**  **(ms/cm)** | **WC**  **(%)** | **clay**  **(%)** | **silt**  **(%)** | **sand**  **(%)** | **SLA**  **(m^2^/kg)** | **LDMC**  **(g/kg)** | **LPC**  **(g/kg)** |
| ROZ | 4.94±5.39 | 7.68±5.90 a | 6.84±3.47 | 52.32±17.25 a | 40.84±20.22 b | 7.67±1.76 | 351.43±82.33 a | 1.59±0.35 b |
| CDZ | 3.80±3.32 | 1.51±1.76 b | 9.18±4.50 | 31.68±17.03 b | 59.14±20.99 a | 6.97±3.64 | 264.07±119.71 b | 2.21±0.51 a |

ROZ, riparian oasis zone; CDZ, circumjacent desert zone; SOC, soil organic carbon; TN, total nitrogen; AN, available nitrogen; TP, total phosphorus; EC, electrical conductivity; WC, soil water content; clay, soil clay content; silt, soil silt content; sand, soil sand content; LT, leaf thickness; SLA, specific leaf area; LDMC, leaf dry matter content; LCC, leaf carbon content; LNC, leaf nitrogen content; LPC, leaf phosphorus content. Data showed “mean value ± standard deviation” that followed by the different letters within each column indicated significant differences between ROZ and CDZ habitats.

| **Table S2. The dominant phylotypes detected at different taxonomical levels** | | | | |
| --- | --- | --- | --- | --- |
| **Different taxonomical level** |  | **Total samples / %** | **CDZ / %** | **ROZ / %** |
| **phylum** |  |  |  |  |
| Proteobacteria | — | 35.0±1.67 | 32.8±1.68 | 37.0±2.71 |
| Actinobacteria | — | 22.0±1.67 | 28.1±2.31 a | 16.9±1.76 b |
| Bacteroidetes | — | 15.2±1.75 | 16.2±3.08 | 14.3±1.94 |
| Firmicutes | — | 6.96±0.91 | 6.37±0.97 | 7.47±1.49 |
| Gemmatimonadetes | — | 6.32±0.86 | 4.26±0.46 b | 8.08±1.45 a |
| Chloroflexi | — | 6.19±0.60 | 6.99±0.79 | 5.51±0.88 |
| Acidobacteria | — | 3.18±0.82 | 0.67±0.12 b | 5.33±1.36 a |
| Deinococcus-Thermus | — | 1.60±0.23 | 2.55±0.28 a | 0.79±0.23 b |
| Verrucomicrobia | — | 0.70±0.12 | 0.33±0.07 b | 1.01±0.18 a |
| Planctomycetes | — | 1.26±0.20 | 0.81±0.18 b | 1.65±0.33 a |
| **genus** |  |  |  |  |
| unidentified |  | 61.1±1.37 | 59.6±1.50 | 62.36±2.2 |
| *Salinimicrobium* | Bacteroidetes | 3.95±0.87 | 4.25±1.40 | 3.70±1.12 |
| *Nafulsella* | Bacteroidetes | 1.55±0.42 | 2.69±0.81 a | 0.57±0.20 b |
| *Pontibacter* | Bacteroidetes | 1.04±0.20 | 1.75±0.32 a | 0.44±0.17 b |
| *Tunicatimonas* | Bacteroidetes | 0.56±0.13 | 1.11±0.23 a | 0.08±0.04 b |
| *Truepera* | Deinococcus-Thermus | 1.60±0.23 | 2.54±0.28 a | 0.79±0.23 b |
| *Rubrobacter* | Actinobacteria | 1.23±0.29 | 2.49±0.49 a | 0.16±0.05 b |
| *Nitrolancea* | Chloroflexi | 0.65±0.16 | 1.10±0.30 a | 0.26±0.08 b |
| *Rubellimicrobium* | Proteobacteria | 0.62±0.19 | 1.24±0.36 a | 0.09±0.04 b |
| *Sphingomonas* | Proteobacteria | 1.03±0.14 | 1.33±0.20 a | 0.77±0.19 b |
| *Marinobacter* | Proteobacteria | 1.51±0.41 | 0.51±0.26 b | 2.37±0.67 a |
| *Marinimicrobium* | Proteobacteria | 0.62±0.17 | 0.15±0.13 b | 1.02±0.26 a |
| *Pseudomonas* | Proteobacteria | 0.78±0.26 | 0.31±0.12 | 1.18±0.45 |
| *Bacillus* | Firmicutes | 1.17±0.17 | 1.36±0.33 | 0.99±0.16 |

Data showed the mean value and standard error of relative abundance in total samples, circumjacent desert zone (CDZ) and riparian oasis zone (ROZ). The different letters at the same row indicated significant differences between CDZ and ROZ habitats.

| **Table S3. One-way analysis of variation of bacterial Shannon index between CDZ and ROZ habitats** | | | | | |
| --- | --- | --- | --- | --- | --- |
|  | **Sum of squares** | **df** | **Mean square** | **F** | **P** |
| **Between groups** | 0.635 | 1 | 0.635 | 0.840 | 0.365 |
| **Within goups** | 27.961 | 37 | 0.756 |  |  |
| **Total** | 28.596 | 38 |  |  |  |

| **Table S4. Correlations between bacterial Shannon index**  **and soil/plant properties** | | | | | |  |
| --- | --- | --- | --- | --- | --- | --- |
|  | **TP (g/kg)** | **EC (ms/cm)** | **silt (%)** | **sand (%)** | **LT (mm)** | |
| Shannon index | -.336 | -.665 | .390 | -.404 | -.326 | |
| P | <0.05 | <0.01 | <0.05 | <0.05 | <0.05 | |

TP, soil total phosphorus; EC, electrical conductivity; silt, soil silt content; sand, soil sand content; LT, leaf thickness.

| **Table S5. Summary statistics of the bacterial β-diversity, as measured with the Bray-Curtis dissimilarity (d_BC_) and its replacement (d_BC-bal_) and nestedness (d_BC-gra_) component.** | | | |
| --- | --- | --- | --- |
|  | **d_BC_** | **d_BC-bal_** | **d_BC-gra_** |
| Value | 0.94 | 0.924 | 0.016 |
| Relative contribution / % |  | 98.335 | 1.665 |

d_BC_, β-diversity; d_BC-bal_, replacement component; d_BC-gra_, nestedness component.

| **Table S6. Explained variation of bacterial β-diversity and its species turnover component based on db-RDA and partial db-RDA analysis** | | | | | | | |
| --- | --- | --- | --- | --- | --- | --- | --- |
| **Effects of** | **Controlling for** | **β-diversity** | | | **species turnover** | | |
|  |  | **adj. R^2^** | **F** | **P** | **adj. R^2^** | **F** | **P** |
| Spatial (S) |  | 0.285 | 6.058 | 0.001 | 0.205 | 4.271 | 0.001 |
| Soil factors (SF) |  | 0.496 | 7.239 | 0.001 | 0.360 | 4.568 | 0.001 |
| Plant community (PC) |  | 0.388 | 5.022 | 0.001 | 0.284 | 3.511 | 0.001 |
| Plant functional traits (PF) |  | 0.272 | 3.846 | 0.001 | 0.210 | 3.026 | 0.001 |
| S+SF+PC+PF |  | 0.686 | 5.146 | 0.001 | 0.498 | 2.887 | 0.001 |
| Spatial (S) | SF+PC+PF | 0.033 | 1.732 | 0.002 | 0.012 | 1.164 | 0.143 |
| Soil factors (SF) | S+PC+PF | 0.072 | 1.921 | 0.001 | 0.060 | 1.480 | 0.001 |
| Plant community (PC) | S+SF+PF | 0.056 | 1.711 | 0.001 | 0.025 | 1.202 | 0.024 |
| Plant functional traits (PF) | S+SF+PC | 0.013 | 1.185 | 0.114 | 0.013 | 1.120 | 0.154 |

| **Table S7. The soil properties that were significantly correlated with bacterial community structure** | | | | | |
| --- | --- | --- | --- | --- | --- |
|  | **d_BC_** | |  | **d_BC-bal_** | |
|  | **r** | **P** |  | **r** | **P** |
| SOC (g/kg) | 0.2573 | 0.0006 |  | 0.2453 | 0.0012 |
| TN (g/kg) | 0.2083 | 0.0047 |  | 0.2272 | 0.0055 |
| AN (mg/kg) | **0.3234** | **0.0001** |  | **0.3252** | **0.0004** |
| TP (g/kg) | 0.1758 | 0.0036 |  | 0.1649 | 0.0097 |
| pH | 0.2019 | 0.0018 |  | 0.2021 | 0.003 |
| EC (ms/cm) | **0.4020** | **0.0001** |  | **0.3430** | **0.0001** |
| WC (%) | **0.4174** | **0.0001** |  | **0.4597** | **0.0001** |
| clay (%) | 0.2034 | 0.0025 |  | 0.223 | 0.0018 |
| silt (%) | **0.3115** | **0.0001** |  | **0.3513** | **0.0001** |
| sand (%) | 0.2804 | 0.0001 |  | 0.3241 | 0.0001 |

SOC, soil organic carbon; TN, soil total nitrogen; AN, soil available nitrogen; TP, soil total phosphorus; EC, soil electrical conductivity; WC, soil water content; clay, (size<2μm); silt, (2μm<size<50μm); sand, (size>50μm).

**Figure legends:**

**Figure S1**. Bacterial community composition across all soil samples at the phylum level. The relative abundance of each taxon was represented using its mean value in all the samples.

**Figure S2**. Relationships between WC, EC, silt, plant richness and bacterial richness (a, b, c, d) or bacterial shannon diversity (e, f, g, h). Changes of bacterial β-diversity and its turnover component with WC (i, m), EC (j, n), silt (k, o) and plant richness pairwise distance (l, p). The fitted lines show regression curves with R^2^ and significance P value.

**Figure S3.** Venn diagram of network nodes between circumjacent desert zone (CDZ) and riparian oasis zone (ROZ).
