# Supplementary figures and images for: Soil Property and Plant Diversity Determine Bacterial Turnover and Network Interactions in a Typical Arid Inland River Basin, Northwest China
Source: Front Microbiol. 2019 Nov 26;10:2655. doi: 10.3389/fmicb.2019.02655 (PMC6888015; doi:10.3389/fmicb.2019.02655)

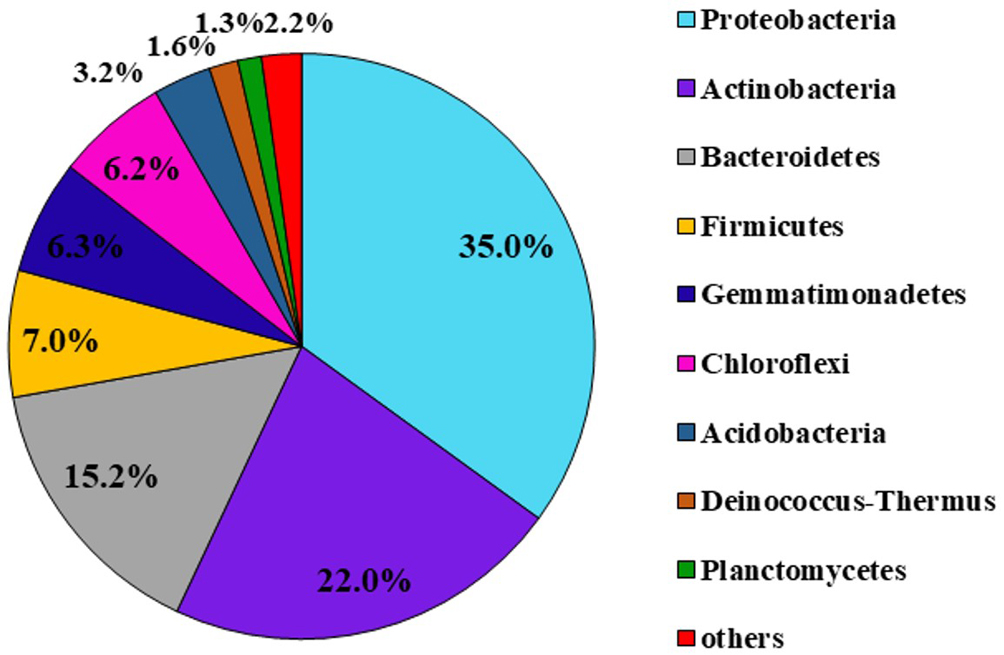

Supplement: Supplementary file 2 [file Image_1.jpg]

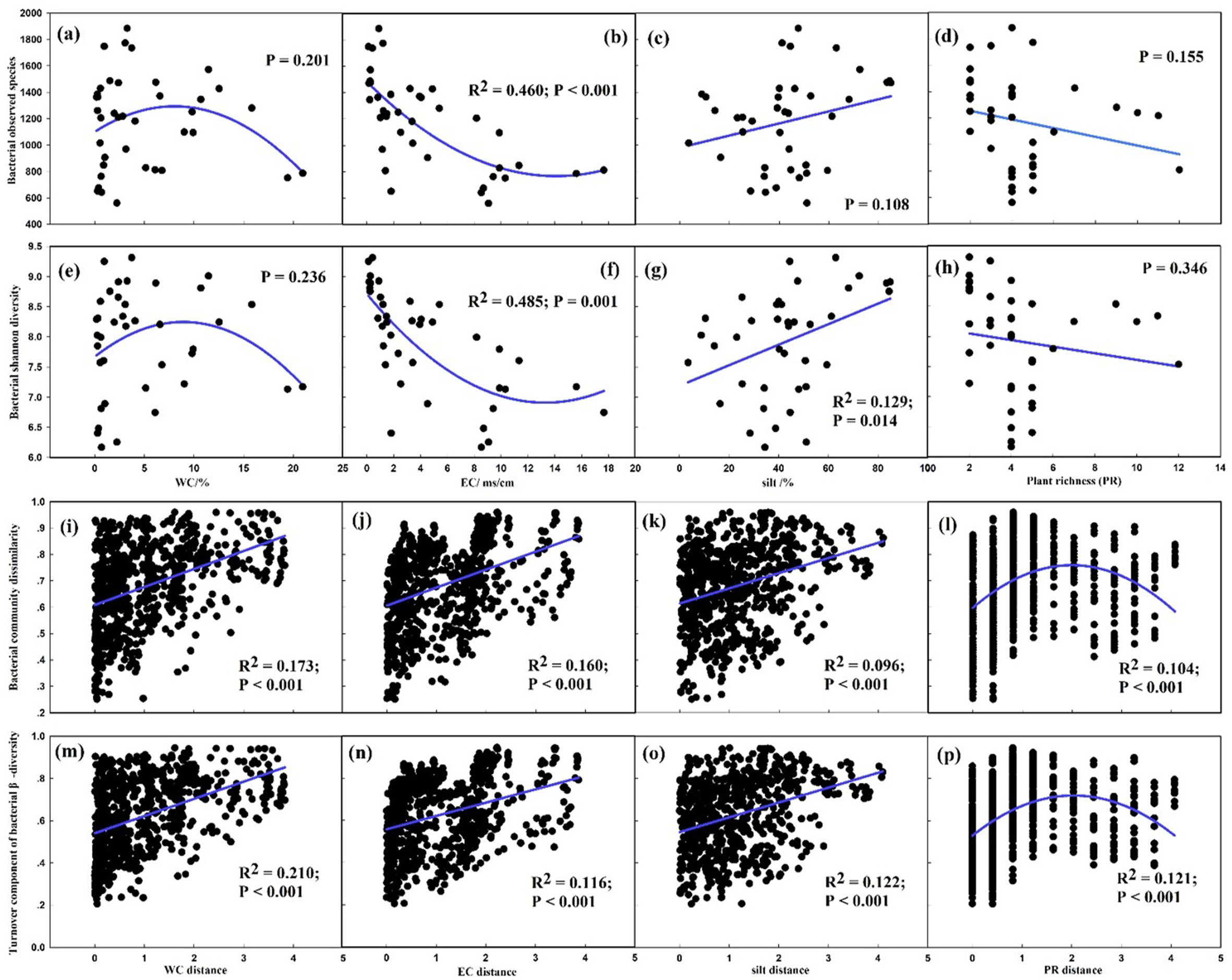

Supplement: Supplementary file 3 [file Image_2.jpg]

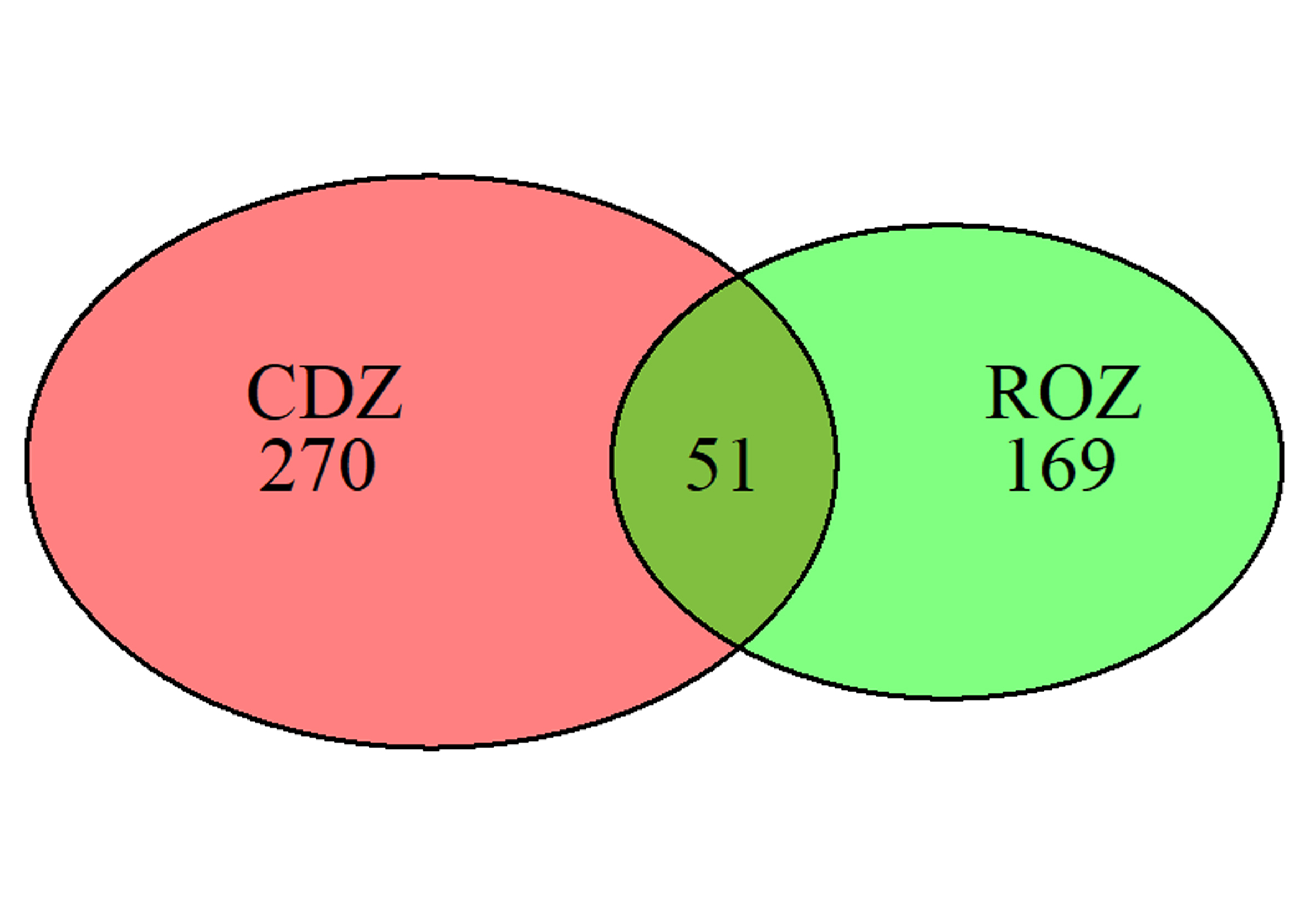

Supplement: Supplementary file 4 [file Image_3.jpg]
